# The Relationship between Trauma Exposure and Psychiatric Hospitalization for Suicide Ideation or Suicide Attempt among Patients Admitted to a Military Treatment Setting

**DOI:** 10.3390/ijerph17082729

**Published:** 2020-04-15

**Authors:** Arthur T. Ryan, Samantha E. Daruwala, Kanchana U. Perera, Su Yeon Lee-Tauler, Jennifer Tucker, Geoffrey Grammer, Jennifer Weaver, Marjan Ghahramanlou-Holloway

**Affiliations:** 1 Veterans Affairs VISN 5 Mental Illness Research, Education and Clinical Center (MIRECC), Baltimore, MD 21201, USA; arthur.ryan@va.gov; 2 Department of Psychiatry, University of Maryland, School of Medicine, Baltimore, MD 21201, USA; 3 Department of Medical and Clinical Psychology, Uniformed Services University of the Health Sciences, Bethesda, MD 20814, USA; samantha.daruwala@usm.edu (S.E.D.); kanchana.perera.ctr@usuhs.edu (K.U.P.); su-yeon.lee-tauler.ctr@usuhs.edu (S.Y.L.-T.); jennifer.a.tucker27.ctr@mail.mil (J.T.); 4 Greenbrook TMS NeroHealth Centers, McLean, VA 22102, USA; geoffrey.grammer@gmail.com; 5 Fort Belvoir Community Hospital, Fort Belvoir, VA 22060, USA; jennifer.j.weaver6.civ@mail.mil

**Keywords:** adverse childhood experience, trauma, suicide, psychiatric hospitalization, military

## Abstract

Suicide attempts and psychiatric hospitalization represent the final outcomes of a complex dynamical system of interacting factors that influence a particular individual’s likelihood of engaging in suicidal behavior, as well as their ability to seek help prior to acting upon suicidal impulses. This study examined the association between different types of lifetime trauma exposure and the likelihood of psychiatric hospitalization following a suicide attempt (SA) rather than suicidal ideation (SI) alone. Electronic medical records for 1100 U.S. military service members and their dependents admitted to a military psychiatric inpatient setting for SA or SI were reviewed for documented lifetime trauma exposure history. Findings indicated that exposure to at least one childhood trauma of any type, and childhood neglect in particular, increased the likelihood that an individual would be hospitalized for SA rather than SI. Exploratory gender-stratified analyses demonstrated that childhood neglect, childhood sexual abuse, and adulthood traumatic loss may be linked with the likelihood of being hospitalized for SA. These findings demonstrate the importance of developing more detailed and nuanced conception of factors known to be associated with suicide as their effects may depend on details of their timing and nature, as well as their interactions with other systems.

## 1. Introduction

Over the past 16 years, the rate of suicide has steadily increased in the United States (U.S.), with the 2018 age-adjusted rate of 14.2 per 100,000 individuals being the highest since 1986 [1,2]. Historically, the suicide rate has been lower among military personnel as compared to age- and sex-adjusted civilians. However, the suicide rate in the military has increased steadily since 2005 and now exceeds that of the age–and sex-adjusted civilian population [3,4]. Suicide is now the leading cause of death in the U.S. military, surpassing accident and combat-related deaths [5]. The increasing suicide rate within the armed forces highlights the need to better understand suicide-associated factors such as lifetime trauma exposure.

Traumatic experiences in childhood, also known as adverse childhood experiences, have shown strong associations with suicidal behavior across the lifespan [6,7,8,9]. Large-scale studies have found childhood trauma exposure increases the odds of suicidal ideation (SI) and suicide attempts (SA) in both children and adults, with higher odds associated with sexual trauma [10]. Additionally, Bruffaerts and colleagues [7] found a strong link between childhood physical and sexual abuse and the onset and persistence of suicidal behavior. The population-attributable risk fraction of lifetime suicide attempts for childhood trauma exposure may be as high as 67% [6]. Similarly, traumatic experiences during adulthood have been associated with suicidal behaviors [11,12]. Even after controlling for a history of childhood trauma, odds for the onset of suicidal behavior increase with adulthood traumas, such as interpersonal violence and sexual assault [12].

Lifetime trauma exposure is also strongly associated with suicide risk in military personnel. In one study of U.S. Army soldiers, 43% of those who died by suicide and 65% of those who made an SA had experienced some form of childhood trauma [13]. Individuals in the Canadian military with a history of childhood abuse also have increased odds of SI and SA [14]. In a sample of active duty U.S. Air Force personnel, SI was associated with unwanted sexual experiences in adulthood, while suicide attempts were associated with rape, robbery, and violent assault in adulthood [15]. Premilitary sexual trauma is associated with increased risk for SI, suicide plans, and SA during one’s military service [16]. Military sexual trauma has been documented as a risk factor for suicide mortality [17].

Several widely used models of suicide conceptualize SI and SA as lying on a continuum of suicidal behavior [18]. Consistent with this, several of the evidence-based practices for reducing the risk of future suicide attempts tacitly assume that SI precedes SA. Suicide safety planning, for example, helps individuals develop a hierarchy of coping strategies to engage in, should they be experiencing thoughts and subsequent impulses to make an SA [19]. Other interventions shown to reduce the risk of future suicide attempts, such as dialectical behavior therapy, also emphasize the development of the ability to inhibit the urge to act upon suicidal impulses and to seek help from emergency services as a final preventative step if the individual is unable to keep himself or herself safe [20]. The characteristics that predispose individuals to seek emergency services prior to engaging in suicidal behavior have been little studied [21]. Some literature suggests that individuals who engage in SI only versus those who make an SA may differ in their impulsivity, aggressiveness, and perhaps other traits [22]. Given the documented link between trauma exposure and SI as well as SA, we were interested to study whether lifetime trauma exposure was associated with the likelihood that an individual would receive psychiatric services for SI alone rather than after they had already made an SA.

The present study aimed to determine whether certain forms of childhood and adulthood trauma exposure increased the likelihood that an individual would be hospitalized for SA rather than for SI. The study was conducted with a sample of military service members and their beneficiaries who were psychiatrically hospitalized for SA or SI. Exposure to childhood and adulthood traumas was expected to increase the likelihood that an individual would be hospitalized for SA rather than SI [22]. Childhood sexual trauma, compared with other lifetime traumas, was hypothesized to most strongly increase the likelihood of being hospitalized for SA rather than SI [10].

## 2. Materials and Methods

### 2.1. Data Collection Procedures

Approval for the study was obtained from the Walter Reed Army Medical Center (WRAMC) and Uniformed Services University of the Health Sciences (USUHS) Institutional Review Boards. The study team was granted access to electronic medical records (EMRs) stored in a standardized electronic record system, Essentris, which is regularly updated with psychiatric inpatient care-related information by medical personnel (e.g., psychiatrists, social workers, and psychiatric nurses) working in the Department of Defense (DoD) treatment facilities. Prior to accessing the data, study coders were trained on patient confidentiality, appropriate use of Essentris, and procedures for finding and extracting information from the EMRs. Study coders were bachelor’s and master’s level research assistants who were trained under the Principal Investigator’s supervision.

### 2.2. Case Selection

Data were collected through a retrospective chart review of EMRs for adult patients (≥18 years) admitted to WRAMC’s psychiatric unit from January 1, 2001 to December 31, 2006. In order to prevent individuals with multiple hospitalizations during the time period from being counted multiple times, individuals with more than one psychiatric hospitalization at WRAMC during the selected time period only had their first episode of hospitalization coded. Data from 333 individuals hospitalized due to SI alone and 767 individuals hospitalized following an SA were collected, yielding a total sample of 1100 unique individuals.

### 2.3. Coding Procedures

Identified EMRs were reviewed and extracted data were entered into a Microsoft Access database. The database included demographics, military service history, suicidal behavior history, documented trauma history, and information pertaining to index hospitalization. The providers who entered this data into the EMR during the course of providing care were not required by hospital policy or otherwise to assess trauma, nor where they required to document the presence or confirmed absence of any trauma type. Thus the medical notes only included information on the presence of a trauma if the provider collected that information and then chose to document it in the medical record. After the EMR was extracted into a database, the trained coders systematically reviewed each individual’s clinical notes using a carefully formulated coding manual and checklist, and noted for each trauma type whether (a) the medical record indicated that the individual had experienced that trauma type or (b) the medical record indicated that either the individual had affirmatively denied experiencing the trauma type or that there was no information at all about the presence or absence of the trauma type. Given that standard practice in the clinical notes was to only note a trauma when it was present and not exhaustively document traumas that had not occurred, trauma types were coded as absent both when the EMR documentation included affirmative denials of the trauma experience and when the EMR documentation lacked any reference to that trauma type. Questions and ambiguities related to coding were discussed and resolved during regularly held research team meetings.

Childhood traumas were categorized into the following types: domestic violence (witnessed by the patient), neglect, physical abuse, sexual abuse, and unspecified trauma. Patients were also rated on the binary variable of “any childhood trauma” based on whether they were exposed to one or more childhood traumas of any type. Adulthood traumas were categorized into the following types: emotional or psychological abuse, military combat, motor vehicle accident, physical assault (other than domestic violence), pregnancy loss (including both abortion and miscarriage for males and females), domestic violence (with the patient as either victim or perpetrator), sexual assault, traumatic loss of a close friend or relative, and unspecified trauma. Note that only in the case of domestic violence were we unable to determine whether the individual was the survivor or the perpetrator of the trauma with the data that were available within the EMR (e.g., only survivors of sexual assault trauma were coded as having a sexual assault trauma). A random sample of 71 EMRs (6.54% of total) was coded by two independent raters. The percentage of agreement between the two raters on each individual trauma type ranged from 78% for childhood observation of domestic violence to 100% for both adulthood pregnancy loss and domestic violence. The average percentage agreement for ratings across all trauma types was 91%.

### 2.4. Data Analysis

We began our analysis by conducting t-tests and chi-squared (*χ*^2^) tests to evaluate differences on demographic variables between the two groups (SA and SI). Second, we compared the SA and SI groups’ prevalence rate for each of the childhood and adulthood traumas individually using chi-square tests. Third, we conducted two separate logistic regressions: the “unadjusted childhood trauma” logistic regression model which included all of the childhood trauma variables as predictors of SA status, and the “unadjusted adulthood trauma” logistic regression model, which included all of the adulthood trauma variables. This allowed us to examine how much unique variance was associated with each of the same-era trauma types. Fourth, we conducted two adjusted logistic regressions: the “adjusted childhood trauma model” and the “adjusted adulthood trauma model.” These adjusted models added demographic variables (i.e., gender, military/civilian status, and age) and a binary variable representing the presence of one or more traumas from the other era (i.e., “any adulthood trauma” to the childhood model and “any childhood trauma” to the adulthood model) as predictors. In the fifth and final step of our analyses, we repeated the adjusted childhood trauma model and the adjusted adulthood trauma model separately for each gender in order to determine whether different types of trauma exposure had different effects depending on the gender of the individual. SAS 9.4 was used for all statistical analyses.

## 3. Results

### 3.1. Demographic Characteristics

Results of tests comparing demographic characteristics in the SA and SI groups are shown in Table 1. Younger age, female gender, lower educational attainment, civilian status, and junior military rank all significantly increased the likelihood that an individual would be hospitalized for SA rather than SI.

### 3.2. Exposure to Individual Childhood and Adulthood Trauma Types

Table 2 shows the results of a comparison between those admitted for SI versus SA in relation to their documented exposure to individual childhood and adulthood trauma types. Exposure to childhood neglect, childhood sexual abuse, or at least one childhood trauma of any type increased the likelihood that an individual would be hospitalized for SA rather than SI. Exposure to adulthood emotional/psychological abuse, adulthood physical assault, and adulthood domestic violence (as victim or perpetrator) increased the likelihood that an individual would be hospitalized for SA rather than SI. Conversely, exposure to adulthood traumatic loss of a friend or family member decreased the likelihood that an individual would be hospitalized for SA rather than SI. This was noteworthy as adulthood traumatic loss was the only form of trauma exposure in our analysis that decreased the likelihood that an individual would be hospitalized for SA.

### 3.3. Unadjusted and Adjusted Childhood Trauma Models

Table 3 displays the results of the unadjusted and adjusted childhood trauma models. The unadjusted childhood trauma model was significant overall: likelihood ratio *χ*^2^ (5, *N* = 1100) = 26.36, *p* < 0.001. Among the individual predictors, both childhood neglect (odds ratio, OR 3.02, 95% confidence interval, CI [1.42, 6.43], *p* = 0.004) and childhood sexual abuse (OR 1.89, 95% CI [1.26, 2.84], *p* = 0.002) significantly increased the likelihood that an individual would be hospitalized for SA rather than SI.

The adjusted childhood logistic regression model was significant overall: likelihood ratio *χ*^2^ (9, *N* = 1100) = 77.83, *p* < 0.001. Among the trauma exposure predictors, childhood neglect (OR 3.08, 95% CI [1.43, 6.63], *p* = 0.004) increased the likelihood that an individual would be hospitalized for SA, while exposure to childhood sexual abuse was no longer significant (OR 1.44, 95% CI [0.95, 2.20], *p* = 0.088).

### 3.4. Unadjusted and Adjusted Adulthood Trauma Models

The unadjusted adulthood trauma model was significant overall: likelihood ratio *χ*^2^ (9, *N* = 1100) = 23.21, *p* = 0.006. Among the individual trauma predictors, only adulthood traumatic loss was significant in predicting decreased likelihood of being hospitalized for SA (OR 0.66, 95% CI [0.46, 0.95], *p* = 0.027).

The adjusted adulthood trauma model was significant overall: likelihood ratio *χ*^2^ (13, *N* = 1100) = 78.81, *p* < 0.001. Among the trauma exposure predictors, exposure to any childhood trauma (OR 1.39, 95% CI [1.01, 1.92], *p* = 0.045) increased the likelihood that an individual would be hospitalized for SA. Exposure to adulthood traumatic loss failed to reach traditional statistical significance in the adjusted model (OR 0.69, 95% CI [0.47, 1.01], *p* = 0.053), though its OR estimate was similar to that found in the unadjusted model.

### 3.5. Exploratory Analyses: Female-Only Adjusted Childhood and Adulthood Trauma Models

The female-only adjusted childhood trauma model was significant overall: likelihood ratio *χ*^2^ (8, *N* = 414) = 25.89, *p* = 0.001. Among the individual trauma predictors, childhood neglect (OR 4.01, 95% CI [1.12, 14.37], *p* = 0.033) significantly increased the likelihood that a female would be hospitalized for SA rather than SI. As compared with the combined male and female sample, the effect of childhood sexual abuse was completely attenuated in the female-only sample (OR 0.86, 95% CI [0.48, 1.58], *p* = 0.644; entire sample OR 1.44, 95% CI [0.95, 2.20], *p* = 0.088).

The female-only adjusted adulthood trauma model was significant overall: likelihood ratio *χ*^2^ (12, *N* = 414) = 30.27, *p* = 0.003. Among the trauma exposure predictors, only adulthood traumatic loss was significant in predicting decreased likelihood of being hospitalized for SA (OR 0.35, 95% CI [0.17, 0.73], *p* = 0.005).

### 3.6. Exploratory Analyses: Male-Only Adjusted Childhood and Adulthood Trauma Models

The male-only adjusted childhood trauma model was significant overall: likelihood ratio *χ*^2^ (8, *N* = 686) = 30.14, *p* < 0.001. Among the trauma exposure predictors, childhood sexual abuse (OR 2.68, 95% CI [1.37, 5.23], *p* = 0.004) significantly increased the likelihood that an individual would be hospitalized for SA rather than SI. Exposure to childhood neglect failed to reach traditional statistical significance (OR 2.62, 95% CI [0.99, 6.92], *p* = 0.052), though its OR estimate was similar to that of childhood sexual abuse.

In regards to the male-only adjusted adulthood trauma model, we noted that the numbers of male participants with documented exposure to adulthood sexual abuse (*n* = 3) and adulthood pregnancy loss in a partner (*n* = 4) were too small to allow for their accurate estimation as predictors in the adjusted adulthood trauma model, and thus these trauma types were excluded from the analysis. The male-only adjusted adulthood trauma model was significant overall: likelihood ratio *χ*^2^ (10, *N* = 686) = 27.47, *p* = 0.002. Among the trauma exposure predictors, only exposure to at least one childhood trauma (OR 1.52, 95% CI [1.02, 2.27], *p* = 0.039) was significantly associated with increased likelihood of being hospitalized for SA rather than SI.

## 4. Discussion

In our analysis of the records of patients psychiatrically hospitalized following suicide-related events, we found several noteworthy findings linking trauma exposure and the likelihood of being hospitalized for SA rather than SI. When considered individually, exposure to childhood neglect, childhood sexual abuse, at least one type of childhood abuse, adulthood emotional/psychological abuse, adulthood physical assault, and adulthood domestic violence all increased the likelihood that an individual would be hospitalized for SA rather than SI. Only adulthood traumatic loss of a friend or relative was found to decrease the likelihood of being hospitalized for SA. When all childhood traumas, demographic variables, and “any adulthood trauma” were entered together, only childhood neglect remained significantly associated with increased likelihood of being hospitalized for SA. When all adulthood traumas, demographic variables, and “any childhood trauma” were entered together, only “any childhood trauma” remained significantly associated with increased likelihood of being hospitalized for SA.

Childhood sexual abuse and adulthood traumatic loss appeared to lose their predictive utility once the effects of demographic factors were adjusted for. However, our single-gender analyses suggested a more interesting possibility, namely that the effects of these trauma types depended upon an individual’s gender. Among female participants, adulthood traumatic loss regained its significant association with a decreased likelihood of admission for SA. Moreover, among females, exposure to any childhood trauma was no longer significantly associated with admission for SA. Among male participants, exposure to childhood sexual abuse and “any childhood trauma” significantly increased the likelihood of being hospitalized for SA rather than SI.

Our analysis suggests that all forms of traumatic experience are not equivalent in affecting the likelihood of being hospitalized for SA rather than SI. Childhood neglect and exposure to one or more childhood trauma types had a reliable relationship with increased likelihood of SA. Our results are consistent with previous research showing that childhood traumatic experiences are more likely than adulthood traumatic experiences to lead to significant and lasting alterations in behavioral, neural, and other biological systems [23]. Childhood trauma exposure is associated with increased aggressiveness, poor interpersonal attachment, impaired problem solving, and increased impulsiveness [24]. Each of these traits have been related to difficulty with regulating the impulse to make an SA while in distress [22].

Childhood trauma exposure, especially childhood sexual abuse, appeared to predispose male individuals to be hospitalized for SA rather than SI. Our findings regarding childhood sexual trauma differ from some previous research, which has shown similar relationships between childhood sexual trauma and suicide attempts in men and women [10]. However, this difference may be explained in light of previous research on the link between childhood sexual trauma and SA in men and our use of a military personnel sample. In a sample of 487 men sexually abused during childhood, Easton, Renner, and O’Leary [25] found that strong identification with traditional western masculinity norms increased the likelihood that the men had made a suicide attempt in the previous 12 months. Male military personnel have been shown to have particularly high levels of identification with traditional masculine norms [26]; thus, it may be the case that childhood sexual trauma is a risk factor for SA specifically among male military personnel. The causal network underlying the identification with traditional masculinity norms and susceptibility to the sequela of childhood sexual trauma is unknown, but may involve reluctance to seek out mental health services [26] and psychological distress associated with traditional masculine norms of heterosexuality and strength clashing with the memories of being sexually assaulted as a child [25]. Female military personnel, in contrast, may show greater resilience to the negative sequelae of childhood trauma and childhood sexual trauma in particular because of their greater willingness to seek help from others, including mental health professionals, and their use of effective coping strategies [27].

The association we found between adulthood traumatic loss of a friend or relative and a decreased likelihood of being hospitalized for SA in females also merits consideration. A large body of research demonstrates that females subjected to major stressful events may engage in the “tend-and-befriend” response [28]. The tend-and-befriend response includes increased attention to interpersonal relationships with friends and family and the active solicitation of help from others. This tend-and-befriend response may make women more likely to seek out emergency psychiatric services prior to engaging in a suicide attempt, either through the encouragement of their family and friends or through increased personal willingness to seek professional assistance. When under stress, men are more likely to engage in the flight-or-fight or avoidance responses [29], which is consistent with our finding that traumatic loss among men was not associated with decreased likelihood of being admitted for SA.

The effect of childhood neglect among females also deserves consideration. Our results are consistent with previous research showing that childhood neglect, particularly emotional neglect, increases the likelihood of future suicide attempts [10]. The apparently larger effect of childhood neglect among female military personnel may have to do with the fact that childhood neglect can impair the ability to form interpersonal attachments and effectively solicit help from others, thus potentially negating the SA-protective social factors that are more common among females.

## 5. Conclusions

In our study of 1100 military personnel, we found that childhood and adulthood traumas predicted the likelihood that an individual would be hospitalized for SA rather than SI. Our findings contribute to the body of literature documenting the connection between trauma exposure and suicidal behavior and have implications for clinical practice and future research studies.

### 5.1. Study Strengths

While several studies have examined the effects of trauma exposure on suicidal behavior in military personnel [13,14,15,16,30,31,32,33,34,35,36,37], our study has several features which set it apart from previous research studies: (1) no previous studies, to our knowledge, have examined a wide variety of childhood and adulthood trauma types in the same population; they have either examined childhood (e.g., [14]) or adulthood (e.g., [31]) traumas or included only a small subset of trauma subtypes from one or the other life stage (e.g., only military sexual trauma [34]); (2) no previous studies to our knowledge have directly compared individuals hospitalized for SI vs. those hospitalized following an SA, an approach which has the advantage of somewhat equalizing the severity of the SI and SA populations (i.e., both had symptoms severe enough to prompt hospitalization) as compared with other studies which have examined history of SI and SA without requiring that both result in hospitalization; (3) many previous studies restricted their samples or the data they collected in several ways that were addressed in our study, for example, by only including suicide attempters (e.g., [13]), by assessing premilitary trauma exposure without separating out childhood and premilitary adulthood trauma exposure (e.g., [16]), and by merging trauma categories that had differential effects in our analyses (e.g., [13], which combined childhood physical, sexual, and emotional abuse into a “personal trauma” category). Overall then, while our study certainly overlaps with and replicates several previous findings documented in the literature studying trauma exposure and suicidal behavior in military personnel, it does provide additional perspective on this complex topic.

### 5.2. Study Limitations

Several important limitations should be considered when interpreting and generalizing from the findings reported here. First, documentation of exposure to different trauma types was ascertained by reviewing medical records produced by clinical personnel. No standardized assessment of trauma exposure was mandated during the time period that this data was collected. Thus, medical personnel were likely to have differed in their likelihood of assessing and documenting the various trauma types we have reported on. If a medical provider did not assess for the presence of a trauma, if they assessed for the trauma and documented its absence, or if they assessed for the trauma but failed to document its presence or absence in the EMR, the trauma would have been rated as absent by the coders when the medical record was reviewed, increasing measurement error, especially for those rated as having the trauma as “absent”. Replication in a cohort of individuals assessed with a standardized trauma interview is thus recommended. However, the fact that our findings are generally consistent with previous studies using more standardized trauma interview measures is reassuring regarding the validity of our data collection methods. Another limitation was that the EMR did not allow us to clearly determine whether the individual was the perpetrator or the survivor of domestic violence trauma. Thus, we were not able to compare the effects of being a perpetrator vs. survivor of domestic violence on suicidal behavior, though previous research has found that both roles in domestic violence are associated with increased risk of suicide attempts [31].

Another limitation of our study was that social stigma against reporting certain forms of trauma exposure (including gender-specific stigma) may have systematically decreased the likelihood of those traumas being reported (e.g., male patients being systematically less willing to report adulthood sexual abuse than female patients). This may have been particularly true in the context of military personnel receiving psychiatric services in a military hospital setting. This reluctance may have contributed to the very low base rate of certain traumas (e.g., adult sexual trauma in males) beyond any true differences in prevalence. However, our results are generally consistent with studies that have employed other methods (e.g., anonymous pen-and-paper surveys) to decrease participants’ reluctance to report stigmatized trauma experiences [25].

### 5.3. Study Recommendations

Future research studies might attempt to replicate our findings using a prospective, prehospitalization military personnel cohort using standardized trauma questionnaires delivered in a manner that increases the likelihood of reporting stigmatized trauma experiences (e.g., pen-and-paper forms not entered into military medical records). Future research should also attempt to distinguish between domestic violence roles (i.e., perpetrator vs. survivor) in order to better understand the effects of one role vs. the other [31]. Future research might also directly measure factors such as impulsivity, adherence to traditional masculine norms, family history of suicidal behavior, tendency to tend-and-befriend, and other factors that might underlie the connection between trauma exposure and later hospitalizations for SI or SA.

In terms of clinical practice implications, outpatient clinicians working with military personnel might gently and thoroughly assess childhood trauma history among their patients. In patients exposed to trauma associated with increased likelihood of being hospitalized for SA rather than SI (e.g., males with childhood sexual trauma), the clinician might attempt to address factors that could reasonably contribute to their patient’s distress and likelihood of not seeking help during a mental health crisis (e.g., unhelpful beliefs regarding male masculinity or difficulty regulating impulsive behaviors when under stress). Particularly in patients with established risk factors for future suicide attempts (e.g., individuals with current SI or who have a history of SA), clinicians might consider using an individual’s trauma history (e.g., a female patient with childhood neglect) to inform their clinical hypothesis about factors that might prevent the individual from seeking help prior to engaging in suicidal behavior (e.g., a lack of strong connections with attentive friends and relatives) and, should those issues be present, targeting them in therapy (e.g., helping to develop appropriate and supportive interpersonal relationships). It is also recommended that hospital admission staff make it standard practice to inquire about previous trauma through structured clinical interviews and consistent documentation methods. Such practices during the admission process will provide more accuracy and better inform risk assessment for suicidality and treatment planning.

## Figures and Tables

**Table 1:** Comparison of demographic characteristics between patients hospitalized for suicide ideation only or following a suicide attempt (*N* = 1100).

Characteristic	Suicide Ideation		Suicide Attempt		Statistics		
	*N* = 333		*N* = 767				
	M	SD	M	SD	*t*(*df*)		*p*-Value
**Age, Years**	28.9	10.7	26.4	8.8	4.01 (1098) **		<0.001
	***n***	**%**	***n***	**%**	***χ*^2^ (*df*)**		***p*-Value**
**Sex**							
Female	84	25.2	330	43.0	31.34 (1)		**<0.001**
Male	249	74.8	437	57.0			
**Race**							
White	203	61	480	62.6	0.26 (1)		0.611
All others	130	39	287	37.4			
**Marital Status**							
Single	149	44.7	388	50.6	3.40 (2)		0.182
Married	130	39.0	275	35.9			
Separated/divorced/widowed	54	16.2	104	13.6			
**Education** ^a^							
High school or equivalent	160	57.4	405	64.5	11.90 (2)		**0.003**
Some college	59	21.2	144	22.9			
Advanced degree	60	21.5	79	12.6			
**Military Status**							
Military	306	91.9	635	82.8	15.56 (1)		**<0.001**
Civilian	27	8.1	132	17.2			
**Paygrade**^a^^							
Junior enlisted (E1–E4)	187	61.5	460	73.3	13.65 (2)		**0.001**
Senior enlisted (E5–E9)	90	29.6	134	21.3			
Warrant officers/officers (W1–O10)	27	8.9	34	5.4			
**Military Branch**^a^^							
Air Force	47	15.4	83	13.1	6.46 (4)		0.167
Army	195	63.9	450	70.9			
Coast Guard	5	1.6	6	0.9			
Marines	26	8.5	34	5.4			
Navy	32	10.5	62	9.8			

*N* = total number; *n* = number of participants; M = mean, SD = standard deviation, *t* = t statistic, *df* = degrees of freedom, *χ*^2^ = chi-squared statistic; *a* Total n’s of the individual categories for this variable may not add up to the total N for suicide ideation or suicide attempt groups because some individuals lacked this information in their electronic medical records (e.g., lacked information on their level of education). ^ Military personnel only.

**Table 2:** Comparison of documented lifetime trauma exposure characteristics between patients hospitalized for suicide ideation only or following a suicide attempt (*N* = 1100).

Trauma Types	Suicide Ideation		Suicide Attempt(s)		Statistics	
	*N* = 333		*N* = 767			
	*n*	%	*n*	%	*χ* ^2^ *(df)*	*p*-Value
Childhood Trauma Type						
Domestic violence	33	9.9	78	10.2	0.02 (1)	0.896
**Neglect**	**9**	**2.7**	**64**	**8.3**	**11.9 (1)**	**0.001**
Physical abuse	65	19.5	175	22.8	1.48 (1)	0.224
**Sexual Abuse**	**39**	**11.7**	**159**	**20.7**	**12.79 (1)**	**<0.001**
Unspecified trauma	12	3.6	47	6.1	2.91 (1)	0.088
**Any childhood Trauma**	**100**	**30.0**	**303**	**39.5**	**8.98 (1)**	**0.003**
Adulthood Trauma Type						
**Domestic violence**	**2**	**0.6**	**21**	**2.7**	**5.18 (1)**	**0.023**
**Emotional/psychological abuse**	**44**	**13.2**	**140**	**18.3**	**4.23 (1)**	**0.040**
Military combat^	31	10.1	62	9.8	0.03 (1)	0.860
Motor vehicle accident	5	1.5	22	2.9	1.81 (1)	0.178
**Physical assault**	**9**	**2.7**	**42**	**5.5**	**4.04 (1)**	**0.045**
Pregnancy loss	2	0.6	16	2.1	3.18 (1)	0.074
Sexual assault	13	3.9	41	5.4	1.03 (1)	0.309
**Traumatic loss**	**59**	**17.7**	**97**	**12.7**	**4.91 (1)**	**0.027**
Unspecified trauma	33	9.9	65	8.5	0.59 (1)	0.443
Any adulthood trauma	142	42.6	340	44.3	0.27 (1)	0.605

*N* = total number; *n* = number of participants; *χ*^2^ = chi-squared statistic, *df* = degrees of freedom, *χ*^2^ = chi-squared statistic; ^ Only military participants included (*N* = 941).

**Table 3:** Odds of psychiatric hospitalization for suicide attempt vs. suicide ideation for patients with each documented lifetime trauma exposure compared to patients without corresponding trauma experience (*N* = 1100).

Trauma Types	Suicide Ideation	Suicide Attempt(s)		
	*N* = 333	*N* = 767	OR (95% CI) ^a^	AOR (95% CI) ^b^
	*n* (%)	*n* (%)		
Childhood Trauma Types			Unadjusted Childhood	Adjusted Childhood
Domestic violence	33 (9.9)	78 (10.2)	0.67 (0.41–1.20)	0.67 (0.40–1.12)
**Neglect**	9 (2.7)	64 (8.3)	3.02 (1.42–6.43) **	3.08 (1.43–6.63) **
Physical abuse	65 (19.5)	175 (22.8)	0.95 (0.66–1.37)	1.02 (0.70–1.49)
**Sexual abuse**	39 (11.7)	159 (20.7)	1.89 (1.26–2.84) **	1.44 (0.95–2.20)
Unspecified trauma	12 (3.6)	49 (6.1)	1.31 (0.65–2.61)	1.34 (0.66–2.72)
Any adulthood trauma	142 (42.6)	340 (44.3)	N/A	0.94 (0.71–1.26)
Adulthood Trauma Types			Unadjusted Adulthood	Adjusted Adulthood
Domestic violence	2 (0.6)	21 (2.7)	3.54 (0.81–15.43)	3.18 (0.72–14.06)
Emotional/psychological abuse	44 (13.2)	140 (18.3)	1.36 (0.94–1.98)	0.97 (0.64–1.48)
Military combat	31 (10.1)	62 (9.8)	0.92 (0.58–1.46)	1.16 (0.72–1.86)
Motor vehicle accident	5 (1.5)	22 (2.9)	1.98 (0.73–5.35)	2.09 (0.76–5.73)
Physical assault	9 (2.7)	42 (5.5)	1.67 (0.78–3.58)	1.52 (0.69–3.37)
Pregnancy loss	2 (0.6)	16 (2.1)	3.30 (0.74–14.73)	2.40 (0.53–10.89)
Sexual assault	13 (3.9)	41 (5.4)	1.10 (0.57–2.14)	0.68 (0.34–1.38)
**Traumatic loss**	59 (17.7)	97 (12.7)	0.66 (0.46–0.95) *	0.69 (0.47–1.01)
Unspecified trauma	33 (9.9)	65 (8.5)	0.84 (0.53–1.32)	0.81 (0.51–1.30)
**Any childhood trauma**	100 (30.0)	303 (39.5)	N/A	1.39 (1.01–1.92) *

*N* = total number, *n* = number of participants, OR = odds ratio, CI = confidence interval; a Unadjusted models do not adjust for age, sex, any adulthood trauma, or any childhood trauma. b Adjusted models controlled for age, sex, and civilian status. The adjusted childhood model also included “any adulthood trauma” as a predictor. The adjusted adulthood model also included “any childhood trauma” as a predictor. * *p* < 0.05. ** *p* < 0.01.
